# Neutralization of Schwann Cell-Secreted VEGF Is Protective to *In Vitro* and *In Vivo* Experimental Diabetic Neuropathy

**DOI:** 10.1371/journal.pone.0108403

**Published:** 2014-09-30

**Authors:** Michela M. Taiana, Raffaella Lombardi, Carla Porretta-Serapiglia, Emilio Ciusani, Norberto Oggioni, Jenny Sassone, Roberto Bianchi, Giuseppe Lauria

**Affiliations:** 1 Neuroalgology and Headache Unit, IRCCS Foundation “Carlo Besta” Neurological Institute, Milan, Italy; 2 Clinical Pathology and Genetics Unit, IRCCS Foundation “Carlo Besta” Neurological Institute, Milan, Italy; 3 Department of Neuroscience and Biomedical Technologies, University of Milan Bicocca, Monza, Italy; Rutgers University, United States of America

## Abstract

The pathogenetic role of vascular endothelial growth factor (VEGF) in long-term retinal and kidney complications of diabetes has been demonstrated. Conversely, little is known in diabetic neuropathy. We examined the modulation of VEGF pathway at mRNA and protein level on dorsal root ganglion (DRG) neurons and Schwann cells (SC) induced by hyperglycaemia. Moreover, we studied the effects of VEGF neutralization on hyperglycemic DRG neurons and streptozotocin-induced diabetic neuropathy. Our findings demonstrated that DRG neurons were not affected by the direct exposition to hyperglycaemia, whereas showed an impairment of neurite outgrowth ability when exposed to the medium of SC cultured in hyperglycaemia. This was mediated by an altered regulation of VEGF and FLT-1 receptors. Hyperglycaemia increased VEGF and FLT-1 mRNA without changing their intracellular protein levels in DRG neurons, decreased intracellular and secreted protein levels without changing mRNA level in SC, while reduced the expression of the soluble receptor sFLT-1 both in DRG neurons and SC. Bevacizumab, a molecule that inhibits VEGF activity preventing the interaction with its receptors, restored neurite outgrowth and normalized FLT-1 mRNA and protein levels in co-cultures. In diabetic rats, it both prevented and restored nerve conduction velocity and nociceptive thresholds. We demonstrated that hyperglycaemia early affected neurite outgrowth through the impairment of SC-derived VEGF/FLT-1 signaling and that the neutralization of SC-secreted VEGF was protective both *in vitro* and *in vivo* models of diabetic neuropathy.

## Introduction

Neuropathy is a chronic complication of both type 1 and type 2 diabetes that severely affects patients’ quality of life and increases morbidity and mortality [1]. Once established, diabetic axonal damage fails to recover due to a number of events, including the loss of innervated targets and the chronic denervation of Schwann cells (SC) [2]. Different mechanisms have been claimed as critical in the pathogenesis of diabetic neuropathy (DN), including abnormal metabolic and neurovascular pathways, growth factor deficiency, and extracellular matrix remodeling [3]. Nevertheless, hyperglycaemia remains the most important trigger for the development of DN and its control is crucial for the course of the disease [3], [4]. The complex relationship between axons and SC in nerve degeneration and regeneration [5] likely plays a critical role also in DN. Previous studies showed that hyperglycaemia can directly affect SC inducing *in vitro* apoptosis [6], altering the secretion of growth factors [7], [8] and interfering with proliferation and migration abilities [9], thus suggesting an effect on this cell type. However, little is known on how hyperglycaemia interferes with the supporting role of SC on axonal growth in cultured dorsal root ganglion (DRG) neurons.

Here we describe that SC mediate the impairment of neurite outgrowth caused by hyperglycaemia through increased secretion of vascular endothelial growth factor (VEGF) and altered fms-related tyrosine kinase 1 (FLT-1) receptor signaling, and that bevacizumab, a molecule that inhibits VEGF activity preventing the interaction to its receptors, *in vitro* prevented axonal outgrowth failure, and *in vivo* both rescued and restored in a dose-dependent fashion DN in rats.

## Materials and Methods

### Animal Experimentation

The Statement of Compliance (Assurance) with Standards for Humane Care and Use of Laboratory Animals has been reviewed (10/28/2008) and approved by the National Institutes of Health-Office for Protection from Research Risks (5023-01, expiration 10/31/2013). The IRCCS Foundation “Carlo Besta” Neurological Institute adheres to the principles set out in the following laws, regulations, and policies governing the care and use of laboratory animals: Italian Legislative Decree 116 of Jan. 27, 1992 Authorization 169/94-A issued Dec. 19, 1994 by Ministry of Health; IRCCS Foundation “Carlo Besta” Institutional Regulations and Policies providing internal authorization for persons conducting animal experiments; the National Institutes of Health *Guide for the Care and Use of Laboratory Animals* (Institute of Laboratory Animal Resources, 1996); and European Union directives and guidelines (Legislative Decree 626, September 19, 1994; 89/391/CEE, 89/654/CEE, 89/655/CEE, 89/656/CEE, 90/269/CEE, 90/270/CEE, 90/394/CEE, 90/679/CEE).

### Cell culture

Primary DRG culture were freshly isolated from embryonic age day-15 Sprague-Dawley rats. Dissected embryonic DRG were enzymatically dissociated with 0.25% trypsin in L-15 medium as previously described [10]. Cells were plated in 24-well plates on collagen-coated glass coverslips, pretreated with poly-D-lysine (Sigma-Aldrich, St. Louis, MO). These cultures contain mainly sensory neurons and SC. DRG neuron monocultures were obtained after exposition to ARA-C (10 µM) for 72 hours [11] and maintained in Neurobasal medium (Gibco Invitrogen, Grand Island, NY) containing 25 mM glucose, supplemented with 1xB27 without antioxidants, penicillin (1 U/L), streptomycin (1 U/L), and nerve growth factor (10 ng/ml). All experiments with DRG culture were performed 7 day after initial plating. SC monocultures were obtained from 2 day-old rat sciatic nerves, purified using a modified Brockes’ method [12], and maintained in DMEM 10% FBS, neuregulin (20 ng/ml) and forskolin (2 µM). Twenty-four hours before the experiments, medium was changed to neuronal medium. Hyperglycemic condition was obtained (where not otherwise specified) adding 20 mM glucose to achieve a final concentration of 45 mM [13], [14]. DRG neuron monocultures were exposed to SC-conditioned medium collected from control and hyperglycemic (45 mM for 24 hours) cultures. Co-cultures and monocultures were exposed to cisplatin or paclitaxel (10 µg/ml and 250 ng/ml for 24 hours, respectively; Sigma-Aldrich, St.Louis, MO) as positive control [15], [16]. Hyperglycemic and control cultures were exposed to bevacizumab (Avastin; Genentech Inc., Roche Group, San Francisco USA), 0.1 and 0.25 mg/ml for 24 hours to evaluate the protective effect [17].

### Assessment of neuronal apoptosis

Cells were fixed and stained with *in* *situ* DNA nick labeling (DeadEnd Fluorometric TUNEL System, Promega, Madison, WI), co-stained with DAPI and anti-BIII tubulin antibody (TuJ1, Berkley Antibody Company, Richmond, CA) and anti-glial fibrillary acidic protein (GFAP, Dako, Glostrup, Denmark) antibodies for neuron and SC identification, respectively. Apoptotic cells were counted using a fluorescence microscope. Apoptosis was also assessed by flow-cytometry using Annexin V/PI assay (Immunostep, Salamanca, Spain) and processed according to manufacturer’s instructions. Early and late apoptosis were evaluated on fluorescence 2 (for propidium iodide) versus fluorescence 1 (for annexin) plots. The percentage of cells stained by annexin V alone was recorded as early apoptosis, whereas the percentage of cells stained by both annexin V and propidium iodide was recorded as late apoptosis.

### Assessment of mitochondrial membrane potential changes

Mitochondrial membrane potential was measured using the J-aggregate forming lipophilic cation (JC-1; Molecular Probes, Eugene, OR) [18], [19]. Dissociated DRG cells were exposed to hyperglycaemia, cisplatin or paclitaxel. After 24 hours, cells were washed and incubated with JC-1 (2.5 µg/ml) for 30 min according to manufacturer’s protocol. The fluorescence emission pattern was analyzed by flow-cytometry using two dimensional green versus red fluorescence plots. The red-to-green ratio allows comparative measurements of membrane potential between cell populations.

### Assessment of axonal outgrowth

After 24 hours exposure to treatments, cells were fixed in 4% paraformaldehyde and stained with fluorescent anti-TuJ1 antibody. Neurite outgrowth was assessed measuring the longest neurite on each of at least 100 randomly selected neurons per condition, using an image analysis system on fluorescence microscope (Image Pro-Plus, Media Cybernetics, Silver Spring, MD) [20]–[22]. Each condition was assessed in duplicate for at least three times using cultures prepared on separate days.

### Rat cytokine array

Cytokine profile expression in SC-conditioned medium was evaluated by Proteome Profiler Rat Cytokine Array Kit, Panel A (R&D Systems, Minneapolis, MN) which detects 29 growth factors, cytokines, and chemokines (CXCL1/CINC-1, IL-1ra, L-Selectin, CXCL3/CINC-2 alpha/beta, IL-2, CXCL9/MIG, CINC-3, IL-3, CCL3/MIP-1 alpha, CNTF, IL-4, CCL20/MIP-3 alpha, Fractalkine/CXC3CL1, IL-6, CCL5/RANTES, GM-CSF, IL-10, CXCL7/Thymus Chemokine, ICAM-1, IL-13, TIMP-1, IFN-gamma, IL-17, TNF-alpha, IL-1 alpha, CXCL10/IP-10, VEGF, IL-1 beta, LIX). Expression was analyzed following manufacturer’s recommendations. The signals were visualized using an ECL system (Amersham Pharmacia Biotech, Piscataway, NJ, USA). Spot densities were measured and compared using Image-J software.

### ELISA assay of secreted VEGF and sFLt-1 protein

Enzyme-linked immunosorbent assay (ELISA) were performed with commercial ELISA kit for VEGF (R&D Systems, Minneapolis, MN) and sFLT-1 (Novatein Biosciences, Cambridge, MA). Conditioned medium was collected from wells 24 hours after treatment. Assays were performed in duplicate and values were compared with standards provided by the kits.

### Real-time PCR assay

Total RNA was isolated 24 hours after treatment with VEGF, glucose and/or bevacizumab from cultured DRG cells or SC using Trizol (Invitrogen, San Giuliano Milanese, Italy) according to manufacturer’s instructions. Precipitated RNA was dissolved in RNase-free water and quantified; aliquots were prepared for further analysis. RNA was analyzed using TaqMan qRT-PCR (quantitative real-time PCR) instrument (CFX384 real time system, Bio-Rad Laboratories, Segrate, Italy) by iScriptTM onestepRT-PCR kit for probes (Bio-Rad Laboratories, Segrate, Italy). Specific TaqMan probes (Life Techonologies, Monza, Italy) were VEGFa (Rn01511601_m1) and VEGFR-1 (Flt1) (Rn00570815_m1). Samples were run in 98 wells in triplicate as multiplexed reactions with a normalizing internal control (18s). Data were confirmed by assay of two additional house-keeping genes: actin (Rn00667869_m1) and cyclophilin A (Rn00690933_m1). Comparative cycle threshold method was used to calculate the relative target gene expression. Each condition was assessed in duplicate for at least three times.

### Western blot

Total proteins from treated cell cultures were extracted using Trizol according to manufacturer’s protocol. Protein quantification was performed by standard Bicinchoninic acid (BCA, Pierce, Rockford, USA) method; 30 µg of protein per lane were applied, separated by electrophoresis on 10% polyacrylamide gels and transferred onto methanol treated PVDF membranes. Membranes were saturated with 5% fat-free dried milk in Tween-triphosphate buffer solution and incubated overnight at 4°C with primary antibodies (anti-VEGF antibody, rabbit policlonal, Abcam, 1∶600; anti-VEGF Receptor 1 antibody, rabbit polyclonal, Abcam 1∶200; anti-alpha tubulin, mouse monoclonal, 1∶800) diluted in the same buffer, washed and exposed to peroxidise conjugated secondary antibodies (donkey anti-rabbit or anti-mouse IgG 1∶10000, GE Healthcare Europe, Milan, Italy). Specific signals were revealed with the chemiluminescence detection kit (ECL, GE Healthcare Europe, Milan, Italy). Semiquantitative densitometric analysis was performed by scanning the bands with image-analysis software (Image-J). Results were normalized for TuJ1.

### Nociceptive thresholds

Thermal nociceptive threshold to radiant heat was quantified measuring withdrawal latency on hot plate [23], defined as the time laps between placement and hindpaw withdrawal and licking. Each animal was tested twice separated by a 30-minute rest interval. Mechanical nociceptive threshold was quantified by the Randall–Selitto paw withdrawal test [23] using an analgesy meter (Ugo Basile, Comerio, Italy) that generates a linearly increasing mechanical force. Result represents the maximal pressure (grams) tolerated by the animal. Rats were accustomed to the devices 3 days before performing the tests. At each time point, animals underwent three trials and values were averaged.

### Electrophysiological techniques

Antidromic sensory tail-nerve conduction velocity (NCV) was assessed by Myto EBNeuro electromyograph (EBNeuro, Firenze, Italy). The latency of potential was recorded in the two sites after nerve stimulation (stimulus duration, 100 msec; filter, 1 Hz to 5 MHz) was determined (peak to peak), and NCV calculated accordingly. Data were collected with a MP100 acquisition system (Biopac Systems, Santa Barbara, CA). All recordings were performed under standard conditions at controlled temperature (room and animals). Core temperature was maintained at 37°C by using heating pads and lamps.

### *In* *vivo* experimental design

**Figure 1:**
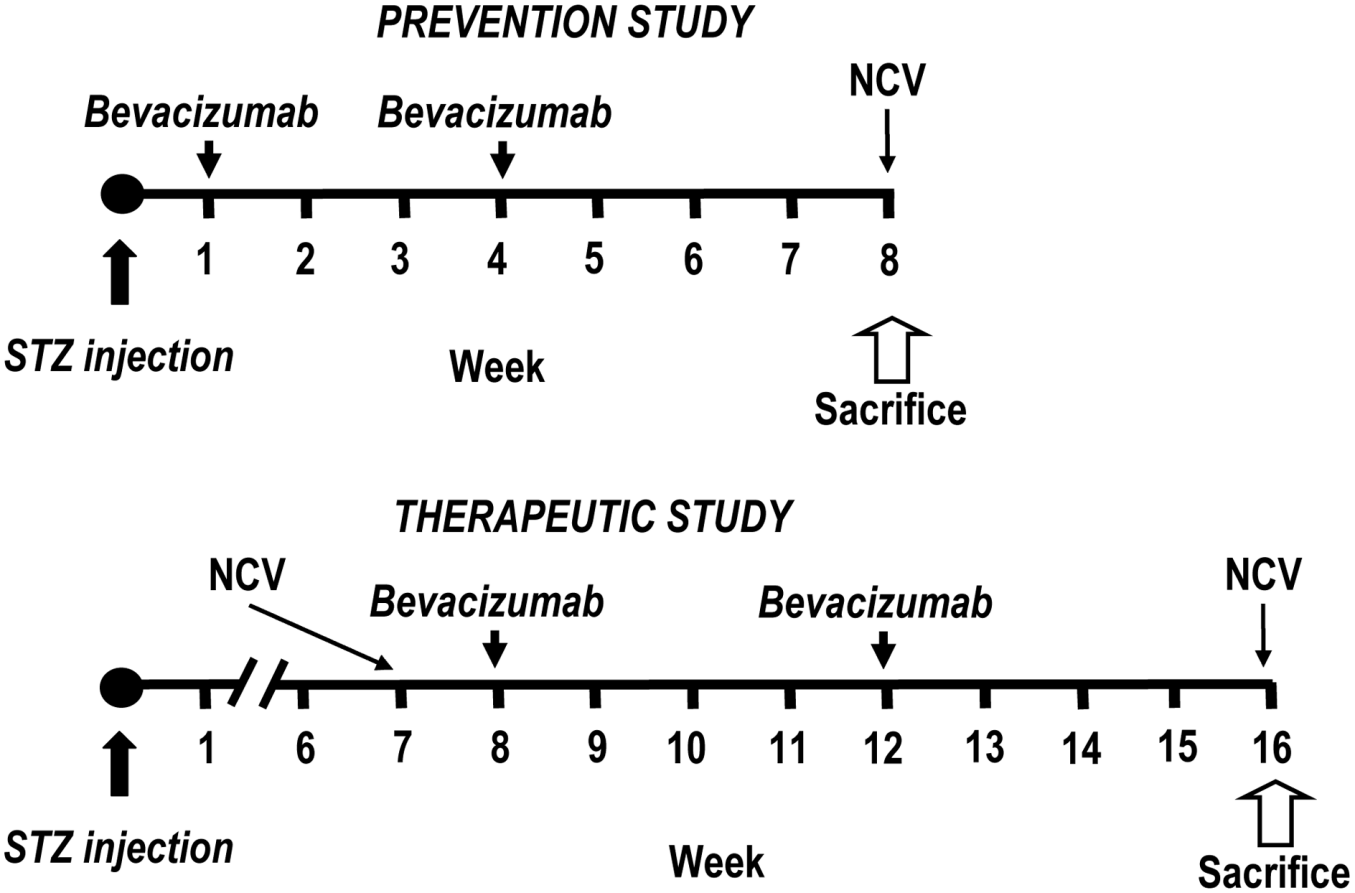
Flow-chart of prevention and therapeutic protocols in streptozotocin-induced diabetic neuropathy. Diabetes was induced by intraperitoneal (i.p.) injection of 60 mg/kg streptozotocin (STZ). Bevacizumab (10 or 20 mg/kg) was administered i.p. at the indicated times. Four groups of Sprague Dawley rats (8 per group) were used in both the protocols (untreated, STZ untreated, STZ+bevacizumab 10 mg/kg, STZ+bevacizumab 20 mg/kg). In the prevention protocol, bevacizumab was administered at week 1 (after confirmation of diabetes), and week 4; rats were sacrificed at week 8. In the therapeutic protocol, bevacizumab was administered at week 8 and 12, and rats were sacrificed at week 16. Nerve conduction velocity (NCV) and nociceptive threshold assessment were measured at the indicated times.

Figure 1 shows the experimental flow-charts. Diabetes was induced in overnight fasted male Sprague Dawley rats weighing 200–220 g (Charles River, Calco, Italy) by single intraperitoneal (i.p.) injection of streptozotocin (STZ, Sigma-Aldrich, St. Louis, MO), 60 mg/kg body weight, in sodium citrate buffer, pH 4.5. Only diabetic rats with urine glucose >15 mM two days after STZ injection were used. Rats were divided into four groups (n = 8) in both the prevention and therapeutic schedules (controls, STZ, STZ+bevacizumab 10 mg/kg, STZ+bevacizumab 20 mg/kg). Control animals were weight, matched and given sodium citrate buffer without STZ. *Prevention protocol:* diabetic rats were randomized to receive i.p. bevacizumab at either 10 or 20 mg/kg body weight at week 1 soon after diabetic induction, and at week 4. NCV and nociceptive thresholds were measured at week 8 (4 weeks after last bevacizumab treatment). *Therapeutic protocol:* diabetic rats underwent NCV and nociceptive threshold assessment at week 7. Therefore, they were randomized to receive bevacizumab at either 10 or 20 mg/kg body weight at week 8 and 12. NCV and nociceptive thresholds were measured at week 16 (4 weeks after last bevacizumab treatment). Body weight was measured weekly in both groups.

### Statistic analysis

Data are presented as mean±standard deviation (SD). Differences were analyzed by Student’s unpaired t test or 1-way ANOVA. P<0.05 was considered statistically significant.

## Results

### Hyperglycaemia did not increase DRG neuron or SC apoptosis

**Figure 2:**
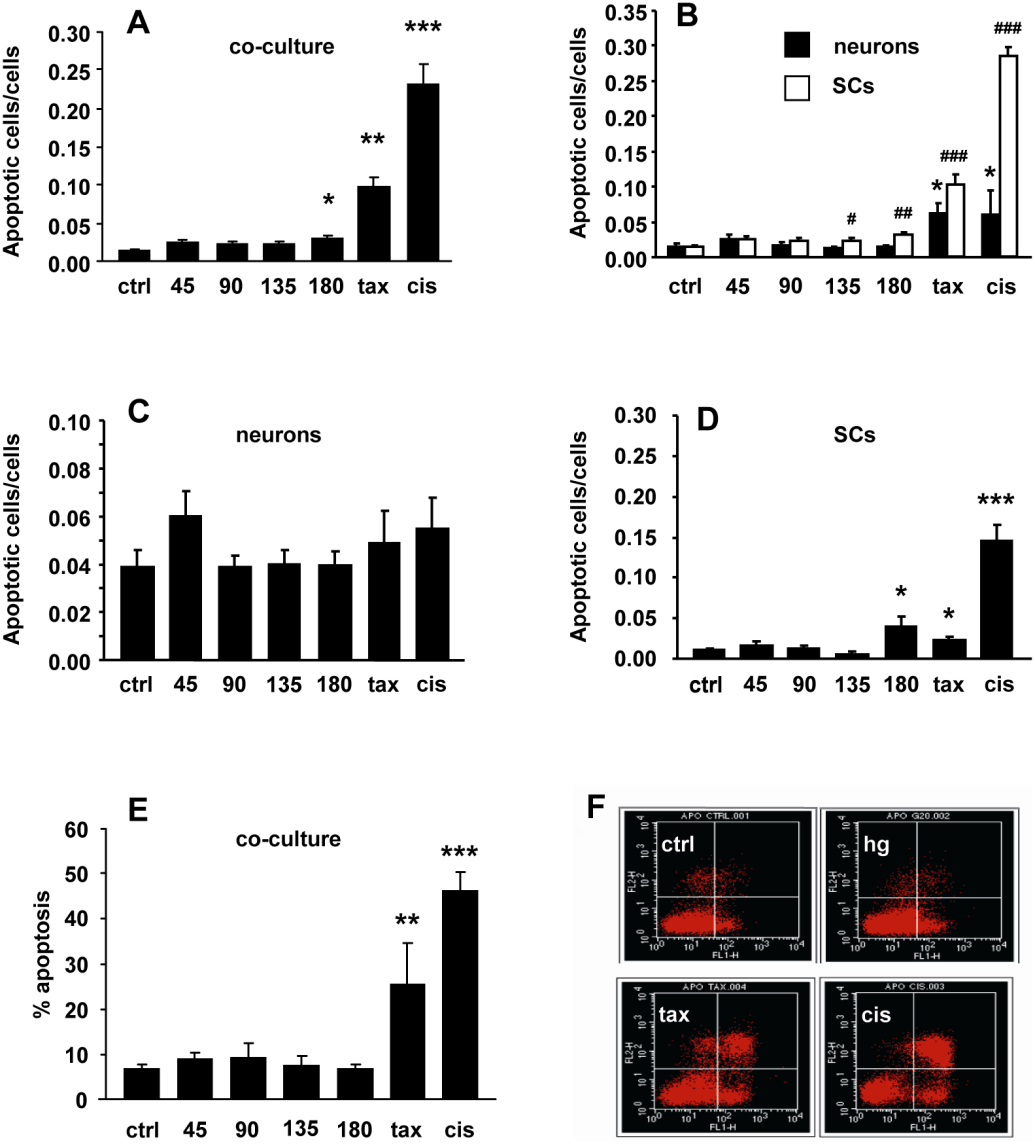
Hyperglycaemia did not increase DRG neuron or Schwann cell (SC) apoptosis. (A) Neuron-SC co-cultures showed a modest increase of apoptosis rate only at the highest glucose concentration; paclitaxel (tax) and cisplatin (cis) exposition was used as positive controls. (B) In DRG neuron monocultures, hyperglycaemia did not induce apoptosis. (C) Tubulin-III and GFAP staining demonstrated that apoptosis mainly involved SC. (D) SC monocultures showed a mild increase of apoptosis rate at the highest glucose concentrations, similar to that observed in co-cultures (see C). (E) Flow cytometry by annexin V/PI assay confirmed the absence of apoptosis in co-cultures exposed to hyperglycaemia. (F) Representative cytogram showing the absence of apoptosis in control co-culture (ctrl) and after 24 hour exposition to hyperglycaemia 45 mM (hg), compared to the high apoptosis rate after exposition to anti-neoplastic compounds (tax, cis). Data are expressed as mean±SEM of independent experiments (*n* = 8) *p<0.05; **p<0.005; ***p<0.0005 vs controls; #p<0.05; ##p<0.005; ###p<0.0005 vs SC controls.

Hyperglycaemia did not significantly affect DRG neuron survival both in coculture (apoptotic cells/cells; ctrl 0.014±0.004, glucose 45 mM 0.024±0.005, glucose 90 mM 0.016±0.003, glucose 135 mM 0.012±0.003, glucose 180 mM 0.014±0.002; **Fig. 2A, B**) and in monoculture (apoptotic cells/cells: ctrl 0.039±0.007, glucose 45 mM 0.060±0.01, glucose 90 mM 0.039±0.005, glucose 135 mM 0.04±0.006, glucose 180 mM 0.04±0.006, **Fig. 2C**) at any of the concentration tested, whereas it increased SC apoptosis only at the highest concentrations both in coculture (apoptotic cells/cells: ctrl 0.015±0.002, glucose 45 mM 0.024±0.004, glucose 90 mM 0.023±0.004, glucose 135 mM 0.023±0.004 p<0.05 vs ctrl, glucose 180 mM 0.031±0.004 p<0.0001 vs ctrl, **Fig. 2B**) and in monoculture (apoptotic cells/cells: ctrl 0.010±0.002, glucose 45 mM 0.016±0.006, glucose 90 mM 0.012±0.004, glucose 135 mM 0.006±0.004, glucose 180 mM 0.031±0.013 p<0.005 vs ctrl, **Fig. 2D**). Conversely, both neurons and SC showed high rate of apoptosis after exposure to cisplatin and paclitaxel demonstrating that absence of intrinsic resistance (apoptotic cells/cells DRG neuron in monoculture ctrl 0.039±0.007, paclitaxel 250 ng/ml 0.049±0.014, cisplatin 10 ug/ml 0.055±0.014; apoptotic cells/cells DRG neuron in coculture ctrl 0.014±0.004, paclitaxel 250 ng/ml 0.061±0.014 p<0.0001 vs ctrl, cisplatin 10 ug/ml 0.06±0.033 p<0.0001 vs ctrl; apoptotic cells/cells SC in monoculture ctrl 0.010±0.002, paclitaxel 250 ng/ml 0.02±0.003 p<0.005 vs ctrl, cisplatin 10 ug/ml 0.145±0.02 p<0.0001 vs ctrl; apoptotic cells/cells SC in coculture ctrl 0.015±0.002, paclitaxel 250 ng/ml 0.101±0.015 p<0.0001 vs ctrl, cisplatin 10 ug/ml 0.284±0.014 p<0.0001 vs ctrl; **Fig. 2A–D**). Flow-cytometry with annexin V/PI confirmed the above described results both for early and late apoptosis (% apoptosis: ctrl 6.3±1.3, glucose 45 mM 8.6±1.5, glucose 90 mM 8.8±3.5, glucose 135 mM 7.0±2.2, glucose 180 mM 6.3±1.3 p<0.005 vs ctrl, paclitaxel 250 ng/ml 25.1±9.2 p<0.005 vs ctrl, cisplatin 10 ug/ml 45.9±4.4 p<0.0001 vs ctrl; **Fig. 2E–F**).

### Hyperglycaemia did not cause early loss of mitochondrial membrane potential differential

**Figure 3:**
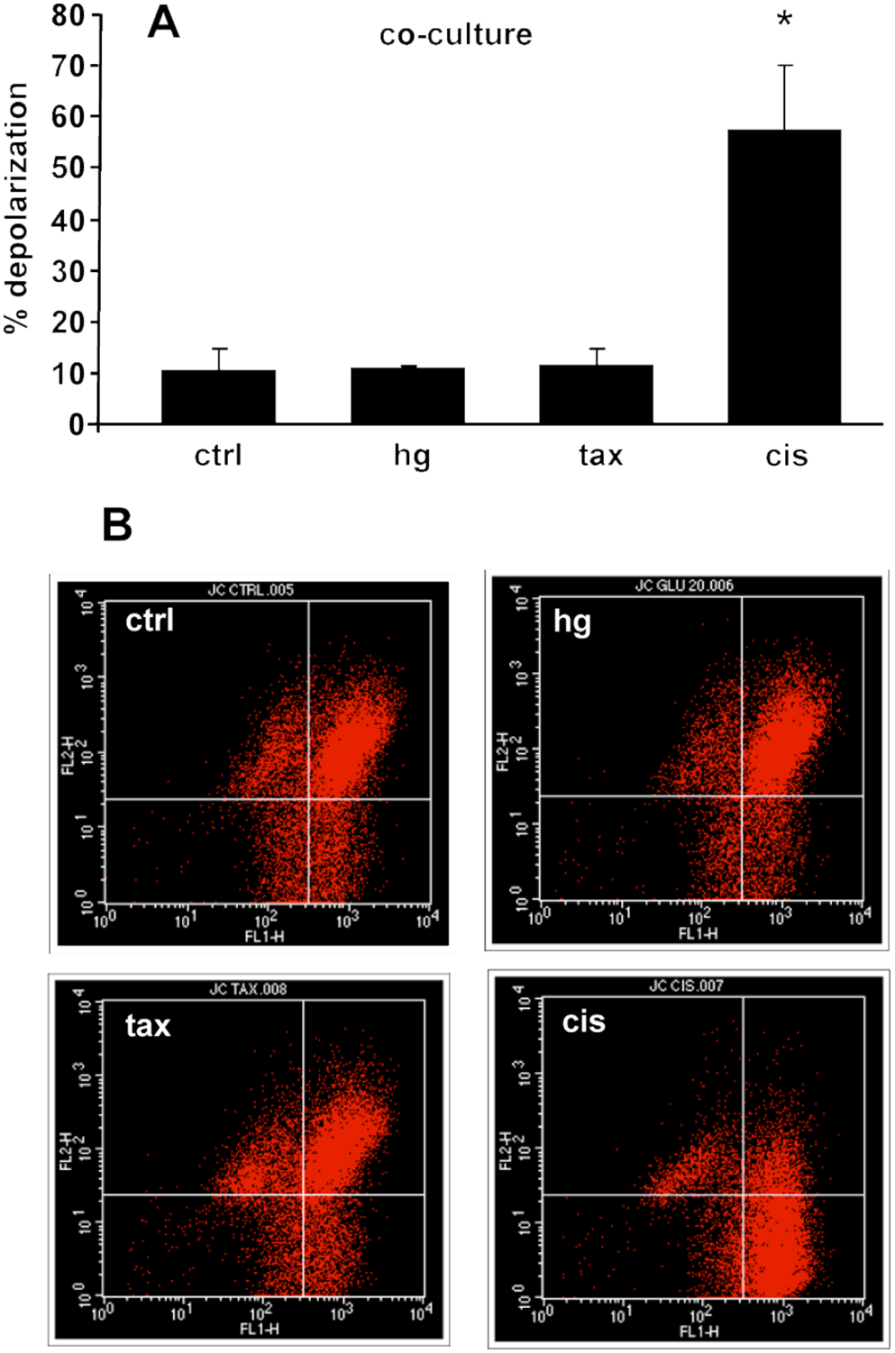
Hyperglycaemia did not affect mitochondrial membrane potential. (A) JC-1 fluorescence emission measurements showed a reduction in red-to-green ratio in neurons exposed to cisplatin (cis) but not to hyperglycaemia (45 mM) nor paclitaxel (tax) (*n* = 4), *p<0.05 vs ctrl. (B) Representative flow cytogram of mitochondrial membrane potential in co-cultures using JC-1. The shift of JC-1 fluorescence from red (FL2) to green (FL1) indicates a collapse of mitochondrial membrane potential.

Mitochondrial potential was assessed in DRG-co-cultures 24 hours after glucose (45 mM) or toxic compound exposure (paclitaxel 250 ng/ml or cisplatin 10 ug/ml). Cisplatin exposure caused an intense decrease of red/green fluorescence ratio (% of cells in the lower-right quadrant 57.40±12.82 vs ctrl 10.65±3.95, p<0.05) thus revealing mitochondrial depolarization. Conversely, high glucose treated cultures did not show any significant change in fluorescence plot (% of cells in the lower-right quadrant 10.82±0.77 vs ctrl 10.65±3.95, p>0.05). These findings indicated that hyperglycaemia did not affect mitochondrial membrane potential in our experimental condition (**Fig. 3A, B**).

### Hyperglycaemia-conditioned SC medium impaired neurite outgrowth and showed high VEGF level

**Figure 4:**
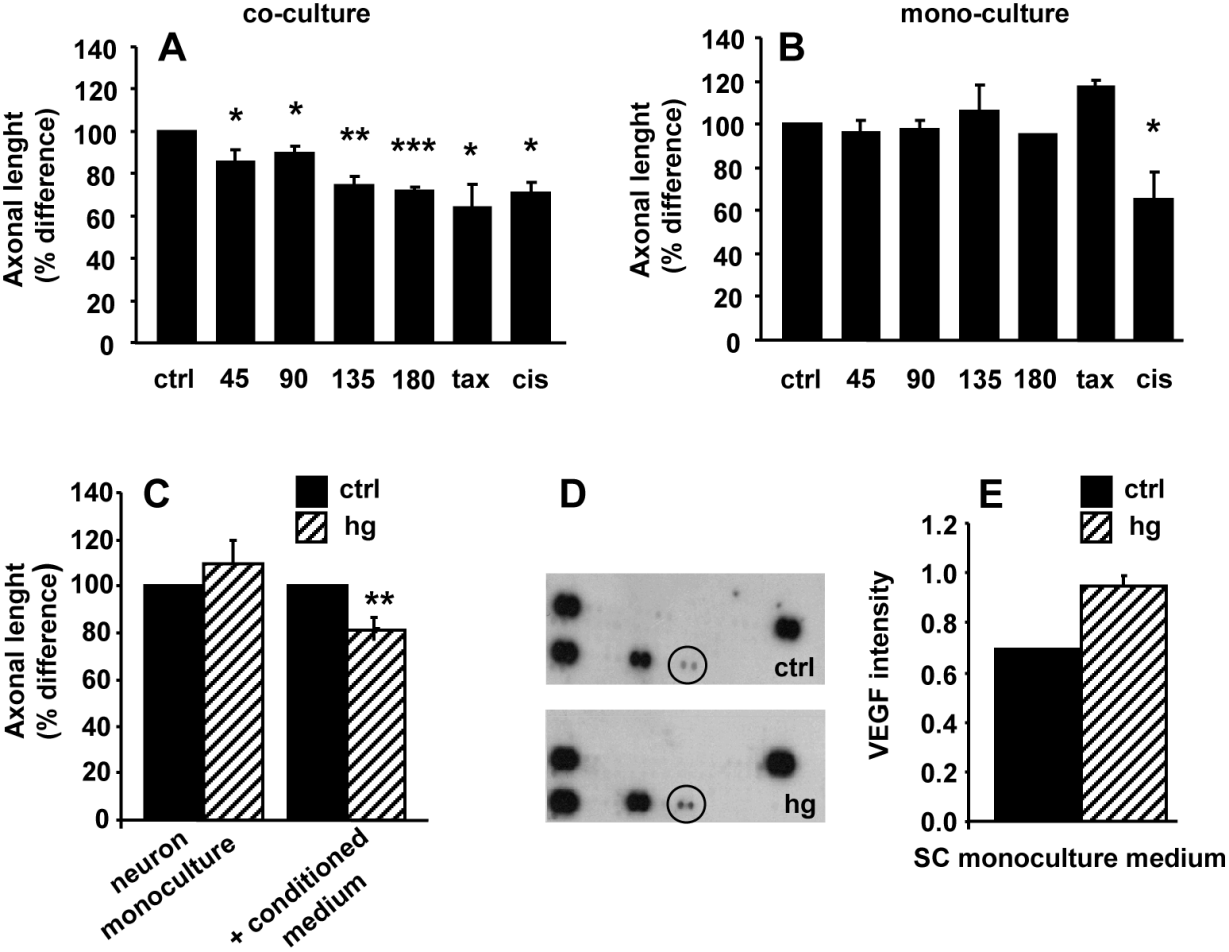
Hyperglycaemia significantly and in a dose-dependent fashion affected neurite outgrowth. (A) Exposure to increasing glucose concentrations induced a significant dose-dependent decrease of neurite outgrowth in neuron-SC co-cultures (B) but not in neuron monocultures. (C) Exposure of neuron monocultures to hyperglycaemia-conditioned SC monoculture medium caused a significant decrease of the neurite outgrowth. All data are normalized to control and presented as mean±SEM of independent experiments (*n* = 8) *p<0.05; **p<0.005; ***p<0.0005 vs control. (D) Cytokine array of hyperglycaemia-conditioned SC monoculture medium showing increase of VEGF compared to control. (E) Data are expressed as relative levels of VEGF.

In DRG co-cultures, 24-hour exposure to increasing hyperglycaemia caused a significant decrease of neurite outgrowth length (axonal length % difference; ctrl 100, glucose 45 mM 85.5±6.2 p<0.05 vs ctrl, glucose 90 mM 89.4±4.0 p<0.05, glucose 135 mM 73.9±4.6 p<0.005 vs ctrl, glucose 180 mM 71.9±1.8 p<0.0001 vs ctrl, paclitaxel 250 ng/ml 63.9±11.6 p<0.05 vs ctrl, cisplatin 10 ug/ml 70.5±5.2 p<0.005 vs ctrl; **Fig. 4A**). Conversely, in DRG neuron monocultures hyperglycaemia did not affect neurite length, suggesting that SC could mediate the neuronal toxicity. This SC-mediated effect appeared hyperglycaemia-specific, because exposure of DRG neuron monocultures to cisplatin caused neurite outgrowth decrease (axonal length % difference: ctrl 100, glucose 45 mM 96.3±5.1, glucose 90 mM 97.3±4.8, glucose 135 mM 106.2±11.7, glucose 180 mM 95.2±4, 9, paclitaxel 250 ng/ml 116.9±17.7, cisplatin 10 ug/ml 65.3±11.2 p<0.05 vs ctrl; **Fig. 4B**). When DRG neuron monocultures were exposed to the medium of SC cultured in hyperglycaemia, neurite outgrowth decreased (axonal length % difference 81.13±1.2 p<0.0001 vs ctrl 100) by similar extent to that observed in DRG co-cultures exposed to hyperglycaemia (axonal length % difference, ctrl 100; glucose 45 mM 109.3±10.4; **Fig. 4C**). These findings suggested that SC secreted a molecule able to exert a toxic effect on DRG neurons. To investigate this hypothesis, we performed a cytokine profile array in the medium of SC monoculture exposed and not exposed to hyperglycaemia. We found higher level of VEGF in the medium of SC monoculture exposed to hyperglycaemia than in control medium (pixel density intensity; ctrl 0.692±0.005; hg 45 mM 0.947±0.050 p<0.05; **Fig. 4D and E**). No difference was found in the levels of all the others cytokines and chemokines analyzed (see Method section for the list of cytokines and chemokines analyzed). This suggested that DRG neurons neurite length decrease may depend on increased level of SC-derived VEGF.

### Hyperglycaemia induced post-translational VEGF regulation in SC

**Figure 5:**
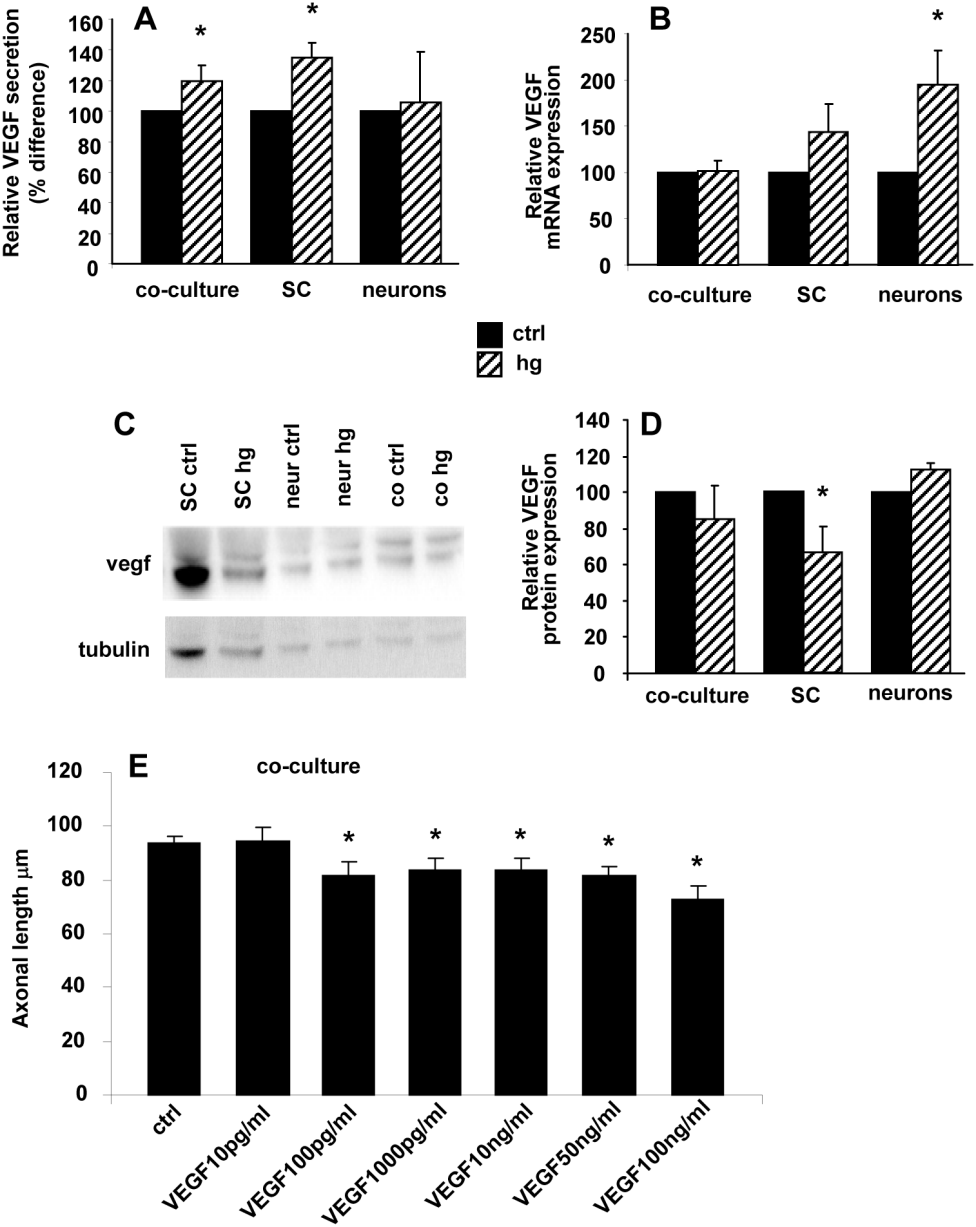
Hyperglycaemia-induced post-translational regulation of VEGF. (A) Exposure to glucose 45 mM for 24 hours increased the level of secreted VEGF in neuron-SC co-cultures and SC monocultures. Data are express as percentage difference between their own control in independent experiments (*n* = 5) *p<0.05. (B) Hyperglycaemia significantly increased VEGF mRNA only in neuron monocultures (*n* = 5). (C) Representative and (D) quantitative western blot (WB) demonstrating VEGF decrease in hyperglycaemia-conditioned SC monocultures compared with control SC monocultures. VEGF level was not affected in neuron monocultures. Data are expressed as mean±SEM of independent experiments (*n* = 4) *p<0.05 vs controls. (E) VEGF induced a significant reduction in axonal outgrowth in neuron/Schwann cells coculture. Data are expressed as mean±SEM of independent experiments (*n* = 3) *p<0.05 vs controls.

We performed ELISA assay for VEGF level in the medium of DRG neuron and SC. Basal VEGF concentration was 14±4 pg/ml in DRG neuron monoculture medium, 3-fold lower than in SC (42±9 pg/ml) and co-culture medium (48±6 pg/ml). After 24 hour exposure to 45 mM glucose, VEGF concentration significantly increased in SC monoculture (45 mM glucose 54±9 pg/ml vs ctrl 42±9 pg/ml, p<0.05) and DRG co-culture (45 mM glucose 56±5 pg/ml vs ctrl 48±6 pg/ml, p<0.05) medium, whereas it did not significantly change in DRG neuron monoculture medium (45 mM glucose 13±4 pg/ml vs ctrl 14±4 pg/ml; **Fig. 5A**). We next performed VEGF mRNA quantification by real-time PCR and VEGF protein quantification by Western blot. After 24 hour exposure to 45 mM glucose, VEGF mRNA expression did not significantly change in SC (relative mRNA expression 144±31 vs ctrl 100), whereas intracellular VEGF protein level decreased (relative protein expression 88.4±13.8 p<0.05 vs ctrl 100). According to the ELISA assay, DRG neuron monocultures in basal condition expressed very low VEGF mRNA level compared to SC (0.004±0.001 vs 0.020±0.01; p<0.01). VEGF mRNA level was significantly increased in DRG neuron monocultures by 24 hour exposure to 45 mM glucose (relative mRNA expression normalized to 18s: 194±37 vs ctrl 100; relative mRNA expression normalized to actin 188±24 vs ctrl 100; relative mRNA expression normalized to cyclophilin A 172±29 vs ctrl 100, p<0.05), whereas intracellular protein level did not change (relative protein expression 113±3 vs ctrl 100) (**Fig. 5B–D**). To test the hypothesis that VEGF decreased neurite outgrowth, DRG neurons were treated for 24 hours with VEGF in a wide range concentration (from 10 pg/ml to 100 ng/ml). VEGF significantly decreased the neurite outgrowth (axonal length µm ctrl 93.8±2.7; VEGF 10 pg/ml 94.5±5.2; VEGF 100 pg/ml 81.7±5.2 p<0.05 vs ctrl; VEGF 1000 pg/ml 83.9±4.2 p<0.05 vs ctrl; VEGF 10 ng/ml 83.3±4.7 p<0.05 vs ctrl; VEGF 50 ng/ml 81.6±3.1 p<0.05 vs ctrl; VEGF 100 ng/ml 72.9±4.7 p<0.05 vs ctrl; **Fig. 5E**), suggesting that VEGF had a key role in the decrease of neurite length found in DRG co-cultures exposed to hyperglycaemia.

### Bevacizumab prevented hyperglycaemia-mediated neurite outgrowth impairment and normalized FLT-1

**Figure 6:**
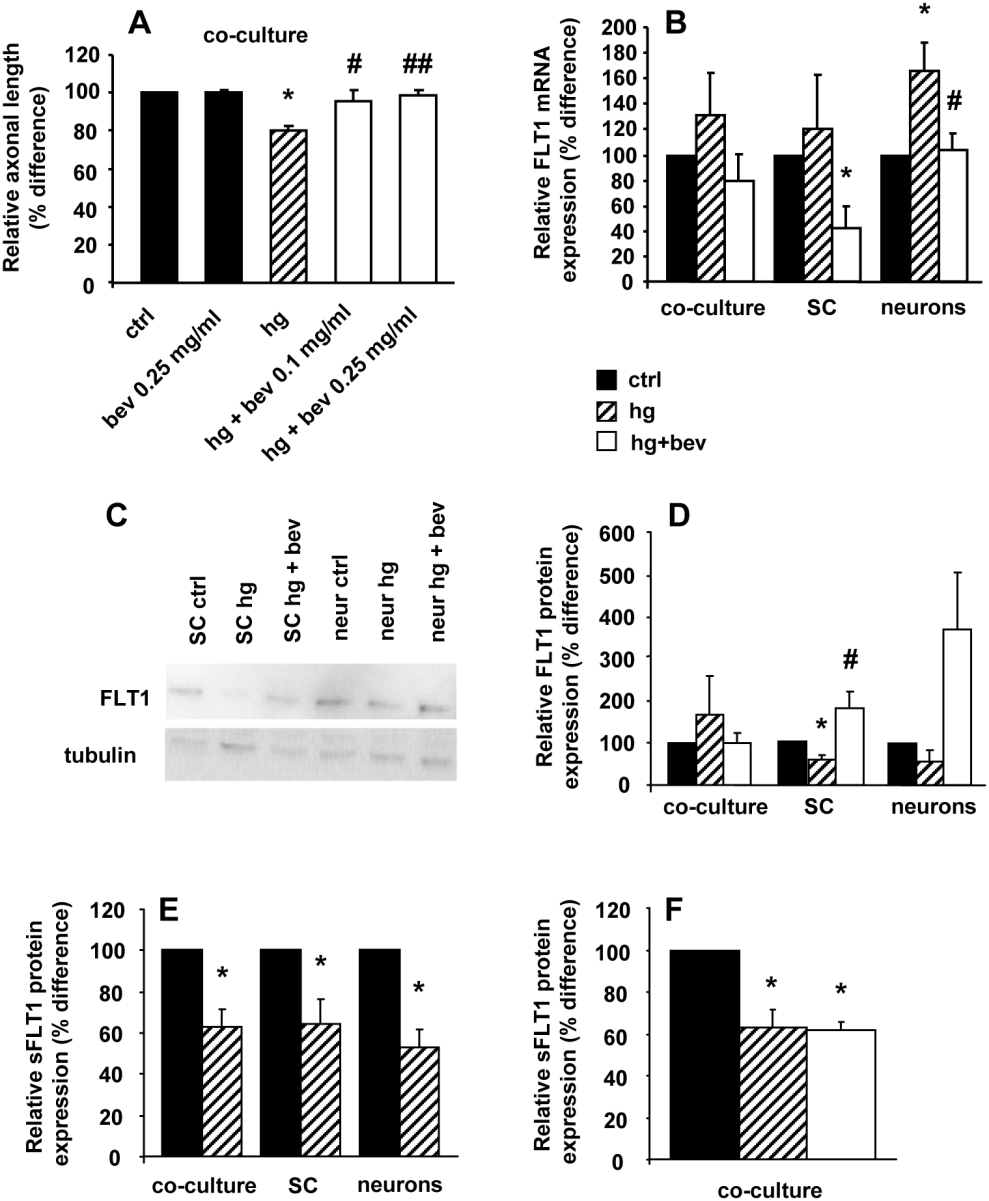
Bevacizumab prevented hyperglycaemia-mediated neurite outgrowth impairment and normalized FLT-1. (A) bevacizumab prevented the decrease of hyperglycaemia-mediated neurite outgrowth in neuron-SC co-cultures (*n* = 5) and (B) the increase of FLT-1 mRNA in neurons monocultures (*n* = 5). (C) Representative and (D) quantitative western blot (WB) (*n* = 5) showing the significant decrease of FLT-1 in hyperglycaemia-conditioned SC monocultures and the preventive effect of bevacizumab. (E) sFLT-1 decreased in the medium of all neuron and SC monocultures and co-cultures exposed to hyperglycaemia (*n* = 5). (F) Bevacizumab did not affect sFLT-1 level in hyperglycaemia-conditioned co-cultures (*n* = 3). Data are expressed as mean±SEM of independent experiments. *p<0.05 vs controls; #p<0.05 vs hyperglycaemia; ##p<0.01 vs hyperglycaemia.

In DRG co-cultures exposed to hyperglycaemia, bevacizumab (0.1 and 0.25 mg/ml) normalized neurite outgrowth length without affecting control cell cultures (axonal length %difference: ctrl 100; bev 0.25 mg/ml 100.4±1.3; hg 80±2.4 p<0.0001 vs ctrl; hg+bev 0.1 mg/ml 95.2±6.3 p<0.05 vs hg; hg+bev 0.25 mg/ml 98.5±3.3 p<0.005 vs hg; **Fig. 6A**). RT-PCR showed that FLT-1 mRNA was constitutively expressed in DRG neurons and SC at low level (0.0020±0.0013 and 0.0015±0.0017, respectively). Hyperglycaemia significantly upregulated FLT-1 mRNA in DRG neuron monocultures (relative expression normalized to 18s 165±23 vs ctrl 100; relative expression normalized to actin 244±0.5 vs ctrl 100; relative expression normalized to cyclophilin A 223±5.1 vs ctrl 100; p<0.05), whereas it did not change its level in DRG co-cultures (131±32 vs ctrl 100) and SC monocultures (121±41 vs ctrl 100). At the same time, hyperglycaemia significantly decreased FLT-1 protein level in SC monocultures (relative protein expression 61±11 vs ctrl 100), whereas FLT-1 protein level remained unchanged in neuron monocultures (55±28 vs ctrl 100) and DRG co-cultures (168±92 vs ctrl 100). Soluble FLT-1 (sFLT-1) protein level decreased after 24 hours exposure to 45 mM glucose both in SC (relative sFLT1 protein expression 64.49±12.0 p<0.05 vs ctrl 100), neuron monoculture (53.3±7.8 p<0.05 vs ctrl 100; mean±SE), and DRG co-culture medium (62.7±8.9 p<0.05 vs ctrl 100; **Fig. 6B–F**). Bevacizumab prevented FLT-1 mRNA upregulation induced by hyperglycaemia in DRG neuron monocultures (relative expression normalized to 18s 103±13 vs hg 165±23; relative expression normalized to cyclophilin A 165±39 vs hg 223±5; p<0.05) and increased FLT-1 protein in SC monocultures (183.6±41.8 p<0.05 vs hg 61±11). sFLT-1 level remained unchanged in DRG co-cultures (61.6±3.9 vs hg 64.49±12.0; **Fig. 6B–F**).

### Bevacizumab improved nerve functions in STZ-diabetic neuropathy rats

**Figure 7:**
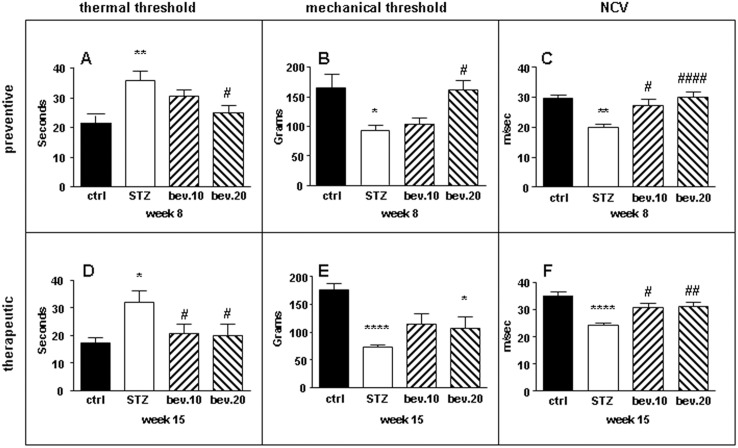
Bevacizumab prevented and restored peripheral nerve functions in diabetic rats. Control and STZ-diabetic rats were treated with bevacizumab according to the prevention (A, B, C) or therapeutic (D, E, F) schedule. (A) Bevacizumab prevented in a dose-dependent fashion thermal hypoalgesia, (B) mechanical threshold decrease and (C) nerve conduction velocity decrease in diabetic rats. In the therapeutic schedule, bevacizumab restored (D) thermal hypoalgesia (E) mechanical threshold and (F) nerve conduction velocity decrease in diabetic rats. Data are expressed as mean±SEM (*n* = 8 animals per group) *p<0.05 vs controls; **p<0.01 vs controls; ****p<0.001 vs controls; #*p<0.05 vs STZ; ##p<0.01 vs STZ.

Bevacizumab did not have significant effects on weight and non-fasted plasma glucose in diabetic rats at both the concentrations administered. In the preventive protocol, bevacizumab prevented the increase of hindpaw thermal nociceptive latency (seconds ctrl 21.4±2.6; STZ 35.8±3.3 p<0.01 vs ctrl; STZ+bev.10 mg/kg 30.5±2.2; STZ+bev.20 mg/kg 27.4±3.4 p<0.05 vs STZ; **Fig. 7A**), the decrease of mechanical thresholds (grams ctrl 164.2±21.3; STZ 94.6±10 p<0.05 vs ctrl; STZ+bev.10 mg/kg 103.8±10.2; STZ+bev.20 mg/kg 162.2±16.2 p<0.05 vs STZ; **Fig. 7B**), and the impairment of NCV in a dose-dependent fashion (m/s ctrl 29.4±0.8; STZ 20.2±0.8 p<0.01 vs ctrl; STZ+bev.10 mg/kg 27.3±1.9 p<0.05 vs STZ; STZ+bev.20 mg/kg 30.0±1.9 p<0.001 vs STZ; **Fig. 7C**) in diabetic rats. In the therapeutic protocol, bevacizumab normalized thermal response latency (seconds ctrl 18.0±2.0; STZ 37.5±2.9 p<0.05 vs ctrl; STZ+bev.10 mg/kg 20.8±3.4 p<0.05 vs STZ; STZ+bev.20 mg/kg 23.4±3 p<0.05 vs STZ; **Fig. 7D**), restored mechanical thresholds (grams ctrl 175.4±10.2; STZ 73.6±3.6 p<0.001 vs ctrl; STZ+bev.10 mg/kg 114.7±18.1; STZ+bev.20 mg/kg 107.2±19.7 p<0.05 vs ctrl; **Fig. 7E**), and rescued NCV (m/s ctrl 34.1±1.7; STZ 24.6±1.1 p<0.001 vs ctrl; STZ+bev.10 mg/kg 29.4±1.8 p<0.05 vs STZ; STZ+bev.20 mg/kg 29.5±2.1 p<0.01 vs STZ; **Fig. 7F**) in diabetic rats.

## Discussion

In patients, DN typically presents as a dying-back axonopathy, suggesting that hyperglycaemia targets axons rather than DRG neurons [3] which primary impairment would lead to distinct non-length-dependent clinical and neurophysiological abnormalities [24]. DRG neurons are particularly vulnerable to toxic circulating agents being supplied by fenestrated capillaries instead of the tight blood-brain-barrier [25], [26]. Furthermore, they have long axons rich in mitochondria and are particularly susceptible to any interference in energy metabolism, oxidative stress, and axonal transport [27]. SC are involved in endogenous neuroprotective pathways [28] and the reciprocal interaction with axons is mandatory for the normal functions of nerves. Previous works showed that hyperglycaemia can impair the secretion of SC-derived cytokines and growth factors, and affect axonal growth [8].

We used an *in* *vitro* experimental approach using co-cultures and monocultures of DRG neurons and SC to investigate the effects of hyperglycaemia on each cell type and their relationship. The use of embryos allows a high content of cells with a higher efficiency in isolating neurons in comparison to post-natal animals and they have been shown to be an adequate and predictive model for studying neuropathies [29]. Even if embryonic sensory neurons have phenotypic differences from adult sensory neurons needing an higher basal concentration of glucose (25 mM) than adult neurons for survival and being dependent on neurotrophic factors [30] for the first week in culture, it has been demonstrated that aged embryonic DRG neurons cultured exhibit properties similar to adult DRG neurons [31]. The equivalent of a 1.8-fold glucose level above control value used was similar to the 1.5-fold increase commonly found in diabetic patients [32]. In this setting, confirming previous works [25], we found that hyperglycaemia affected the ability of neurite outgrowth, whereas it did not induce early or late DRG neuron apoptosis. Although neurite outgrowth is a model of axon elongation rather than degeneration, our findings support what has been clearly established in animal models and patients with DN [3] and points to axons as the primary target of hyperglycaemia-induced toxicity.

Our study demonstrated the pathogenetic role of SC-derived VEGF/FLT-1 signaling, whose normalization after bevacizumab treatment was protective in the *in* *vitro* model. We showed that the exposition of DRG neurons monoculture to hyperglycaemia did not affect neurite outgrowth, which did not differ from control monocultures. Conversely, neurite outgrowth significant decreased when DRG neuron monocultures were exposed to the medium of SC cultured in hyperglycaemia. This effect was mediated by the marked increase of VEGF in the medium of hyperglycaemia-conditioned SC monoculture, as confirmed by the dose-dependent impairment of neurite outgrowth after exposition of DRG co-culture to VEGF. Previous studies showed that hyperglycaemia directly stimulates the secretion of VEGF in retinal Müller cells [33] and proximal tubular cells [34]. Moreover it has been observed that VEGF protein level increased in DRG neurons and sciatic nerve axons from chronic STZ diabetic rats [35]. These findings strengthened the hypothesis of a key role of VEGF also in the pathogenesis of DN, like already demonstrated in diabetic retinopathy and nephropathy [36], [37].

We demonstrated that hyperglycaemia inversely modified FLT-1 protein level in DRG neuron monocultures (where it increased) and SC (where it decreased). FLT-1 is a cell-surface receptor for VEGF and may function as negative regulator limiting the amount of free VEGF and preventing its binding to VEGF receptor-2 (KDR), the best characterized receptor and known to mediate most VEGF cellular responses [38]. We also found that sFLT-1 was decreased. This soluble receptor lacks one transmembrane domain and may function as a decoy for VEGF. We speculated that in hyperglycaemia VEGF overruled sFLT-1 whose scavenger activity could not limit VEGF increase and its toxic effects.

We showed that bevacizumab, a recombinant humanized monoclonal IgG1 antibody that binds VEGF and inhibits its biologic activity preventing the interaction to its receptors, was protective both *in* *vitro* and *in* *vivo* models of DN. Indeed, it reduced the level of free VEGF in the medium of DRG co-cultures exposed to hyperglycaemia and protected from the impairment of neurite outgrowth in a dose-dependent fashion. This was associated with the normalization of FLT-1 signaling between neurons and SC. Exposition of hyperglycaemia-conditioned DRG neuron monocultures to bevacizumab normalized FLT-1 mRNA with no change at the protein level. Conversely, exposition of hyperglycaemia-conditioned SC monocultures to bevacizumab increased FLT1 protein and reduced FLT-1 mRNA.

Finally, we demonstrated that bevacizumab both protected and reversed neuropathy in STZ rats, confirming the neuroprotective effects of our *in* *vitro* studies. Indeed, preventive and therapeutic protocols of bevacizumab administration counteracted in a dose-dependent fashion the pathological changes in thermal and mechanical thresholds, and in NCV which are hallmarks of diabetic neuropathy. Modulation of the VEGF/FLT1 signalling axis *in* *vivo* have to be further investigate in order to attribute efficacy of bevacizumab to a specific mechanism. Few and contradictory data are available on the role of VEGF in DN. Some works showed neuroprotective effects of VEGF on sensory and motor neurons [39]–[41], whereas others provided convincing evidence of direct toxic effects on nerves [42] which are reversed by bevacizumab [43]. A recent study reported significantly higher levels of serum VEGF in diabetic patients with neuropathy compared to those without neuropathy [44]. In the STZ model of experimental diabetes it has been reported that VEGF expression increased in DRG and sciatic nerves and that insulin and/or nerve growth factor administration could prevent it [35].

## Conclusions

We provided evidence of the pathogenetic role of VEGF in experimental DN. Our findings are in keeping with previous studies showing the direct involvement of VEGF in other long-term complications of diabetes, as demonstrated by its detrimental effects on retina [36] and kidney [45], the elevated serum levels [37], [46] and, most importantly, the beneficial effects of anti-VEGF treatment in patients with diabetic retinopathy and nephropathy [47]–[50]. We suggest that appropriate clinical trials should be designed to test the efficacy of anti-VEGF drugs also in DN patients.

## References

[1] Said G (2007) Diabetic neuropathy–a review. Nat Clin Pract Neurol 3: 331–340. 17549059 10.1038/ncpneuro0504

[2] Kennedy JM, Zochodne DW (2005) Impaired peripheral nerve regeneration in diabetes mellitus. J Peripher Nerv Syst 10: 144–157. 15958126 10.1111/j.1085-9489.2005.0010205.x

[3] Zochodne DW (2007) Diabetes mellitus and the peripheral nervous system: manifestations and mechanisms. Muscle Nerve 36: 144–166. 17469109 10.1002/mus.20785

[4] listed Na (1995) The effect of intensive diabetes therapy on the development and progression of neuropathy. The Diabetes Control and Complications Trial Research Group. Ann Intern Med 122: 561–568. 7887548 10.7326/0003-4819-122-8-199504150-00001

[5] Sulaiman OA, Gordon T (2000) Effects of short- and long-term Schwann cell denervation on peripheral nerve regeneration, myelination, and size. Glia 32: 234–246. 11102965 10.1002/1098-1136(200012)32:3<234::aid-glia40>3.0.co;2-3

[6] Leinninger GM, Vincent AM, Feldman EL (2004) The role of growth factors in diabetic peripheral neuropathy. J Peripher Nerv Syst 9: 26–53. 14871451 10.1111/j.1085-9489.2004.09105.x

[7] Delaney CL, Russell JW, Cheng HL, Feldman EL (2001) Insulin-like growth factor-I and over-expression of Bcl-xL prevent glucose-mediated apoptosis in Schwann cells. J Neuropathol Exp Neurol 60: 147–160. 11273003 10.1093/jnen/60.2.147

[8] Tosaki T, Kamiya H, Yasuda Y, Naruse K, Kato K, et al (2008) Reduced NGF secretion by Schwann cells under the high glucose condition decreases neurite outgrowth of DRG neurons. Exp Neurol 213: 381–387. 18675804 10.1016/j.expneurol.2008.06.017

[9] Gumy LF, Bampton ET, Tolkovsky AM (2008) Hyperglycaemia inhibits Schwann cell proliferation and migration and restricts regeneration of axons and Schwann cells from adult murine DRG. Mol Cell Neurosci 37: 298–311. 18024075 10.1016/j.mcn.2007.10.004

[10] Fex Svenningsen A, Shan WS, Colman DR, Pedraza L (2003) Rapid method for culturing embryonic neuron-glial cell cocultures. J Neurosci Res 72: 565–573. 12749021 10.1002/jnr.10610

[11] Melli G, Keswani SC, Fischer A, Chen W, Hoke A (2006) Spatially distinct and functionally independent mechanisms of axonal degeneration in a model of HIV-associated sensory neuropathy. Brain 129: 1330–1338. 16537566 10.1093/brain/awl058

[12] Brockes JP, Fields KL, Raff MC (1979) Studies on cultured rat Schwann cells. I. Establishment of purified populations from cultures of peripheral nerve. Brain Res 165: 105–118. 371755 10.1016/0006-8993(79)90048-9

[13] Leinninger GM, Backus C, Sastry AM, Yi YB, Wang CW, et al (2006) Mitochondria in DRG neurons undergo hyperglycemic mediated injury through Bim, Bax and the fission protein Drp1. Neurobiol Dis 23: 11–22. 16684605 10.1016/j.nbd.2006.01.017

[14] Chattopadhyay M, Walter C, Mata M, Fink DJ (2009) Neuroprotective effect of herpes simplex virus-mediated gene transfer of erythropoietin in hyperglycemic dorsal root ganglion neurons. Brain 132: 879–888. 19244253 10.1093/brain/awp014 PMC2724909

[15] Wang MS, Davis AA, Culver DG, Wang Q, Powers JC, et al (2004) Calpain inhibition protects against Taxol-induced sensory neuropathy. Brain 127: 671–679. 14761904 10.1093/brain/awh078

[16] Ta LE, Espeset L, Podratz J, Windebank AJ (2006) Neurotoxicity of oxaliplatin and cisplatin for dorsal root ganglion neurons correlates with platinum-DNA binding. Neurotoxicology 27: 992–1002. 16797073 10.1016/j.neuro.2006.04.010

[17] Luthra S, Narayanan R, Marques LE, Chwa M, Kim DW, et al (2006) Evaluation of in vitro effects of bevacizumab (Avastin) on retinal pigment epithelial, neurosensory retinal, and microvascular endothelial cells. Retina 26: 512–518. 16770256 10.1097/01.iae.0000222547.35820.52

[18] Kulkarni GV, Lee W, Seth A, McCulloch CA (1998) Role of mitochondrial membrane potential in concanavalin A-induced apoptosis in human fibroblasts. Exp Cell Res 245: 170–178. 9828113 10.1006/excr.1998.4245

[19] Smiley ST, Reers M, Mottola-Hartshorn C, Lin M, Chen A, et al (1991) Intracellular heterogeneity in mitochondrial membrane potentials revealed by a J-aggregate-forming lipophilic cation JC-1. Proc Natl Acad Sci U S A 88: 3671–3675. 2023917 10.1073/pnas.88.9.3671 PMC51514

[20] Melli G, Taiana M, Camozzi F, Triolo D, Podini P, et al (2008) Alpha-lipoic acid prevents mitochondrial damage and neurotoxicity in experimental chemotherapy neuropathy. Exp Neurol 214: 276–284. 18809400 10.1016/j.expneurol.2008.08.013

[21] Zhang G, Lehmann HC, Manoharan S, Hashmi M, Shim S, et al (2011) Anti-ganglioside antibody-mediated activation of RhoA induces inhibition of neurite outgrowth. J Neurosci 31: 1664–1675. 21289175 10.1523/JNEUROSCI.3829-10.2011 PMC3758521

[22] Liu R, Lin G, Xu H (2013) An efficient method for dorsal root ganglia neurons purification with a one-time anti-mitotic reagent treatment. PLoS One 8: e60558. 23565257 10.1371/journal.pone.0060558 PMC3614500

[23] Bianchi R, Cervellini I, Porretta-Serapiglia C, Oggioni N, Burkey B, et al (2012) Beneficial effects of PKF275-055, a novel, selective, orally bioavailable, long-acting dipeptidyl peptidase IV inhibitor in streptozotocin-induced diabetic peripheral neuropathy. J Pharmacol Exp Ther 340: 64–72. 21984837 10.1124/jpet.111.181529

[24] Sghirlanzoni A, Pareyson D, Lauria G (2005) Sensory neuron diseases. Lancet Neurol 4: 349–361. 15907739 10.1016/S1474-4422(05)70096-X

[25] Zochodne DW, Verge VM, Cheng C, Sun H, Johnston J (2001) Does diabetes target ganglion neurones? Progressive sensory neurone involvement in long-term experimental diabetes. Brain 124: 2319–2334. 11673332 10.1093/brain/124.11.2319

[26] Akude E, Zherebitskaya E, Roy Chowdhury SK, Girling K, Fernyhough P (2010) 4-Hydroxy-2-nonenal induces mitochondrial dysfunction and aberrant axonal outgrowth in adult sensory neurons that mimics features of diabetic neuropathy. Neurotox Res 17: 28–38. 19557324 10.1007/s12640-009-9074-5 PMC2894940

[27] Zherebitskaya E, Akude E, Smith DR, Fernyhough P (2009) Development of selective axonopathy in adult sensory neurons isolated from diabetic rats: role of glucose-induced oxidative stress. Diabetes 58: 1356–1364. 19252136 10.2337/db09-0034 PMC2682687

[28] Hoke A (2006) Mechanisms of Disease: what factors limit the success of peripheral nerve regeneration in humans? Nat Clin Pract Neurol 2: 448–454. 16932603 10.1038/ncpneuro0262

[29] Melli G, Hoke A (2009) Dorsal Root Ganglia Sensory Neuronal Cultures: a tool for drug discovery for peripheral neuropathies. Expert Opin Drug Discov 4: 1035–1045. 20657751 10.1517/17460440903266829 PMC2908326

[30] Lindsay RM (1988) Nerve growth factors (NGF, BDNF) enhance axonal regeneration but are not required for survival of adult sensory neurons. J Neurosci 8: 2394–2405. 3249232 10.1523/JNEUROSCI.08-07-02394.1988 PMC6569525

[31] Yu C, Rouen S, Dobrowsky RT (2008) Hyperglycemia and downregulation of caveolin-1 enhance neuregulin-induced demyelination. Glia 56: 877–887. 18338795 10.1002/glia.20662 PMC2553896

[32] Stumvoll M, Goldstein BJ, van Haeften TW (2005) Type 2 diabetes: principles of pathogenesis and therapy. Lancet 365: 1333–1346. 15823385 10.1016/S0140-6736(05)61032-X

[33] Schrufer TL, Antonetti DA, Sonenberg N, Kimball SR, Gardner TW, et al (2010) Ablation of 4E-BP1/2 prevents hyperglycemia-mediated induction of VEGF expression in the rodent retina and in Muller cells in culture. Diabetes 59: 2107–2116. 20547975 10.2337/db10-0148 PMC2927931

[34] Katavetin P, Miyata T, Inagi R, Tanaka T, Sassa R, et al (2006) High glucose blunts vascular endothelial growth factor response to hypoxia via the oxidative stress-regulated hypoxia-inducible factor/hypoxia-responsible element pathway. J Am Soc Nephrol 17: 1405–1413. 16597689 10.1681/ASN.2005090918

[35] Samii A, Unger J, Lange W (1999) Vascular endothelial growth factor expression in peripheral nerves and dorsal root ganglia in diabetic neuropathy in rats. Neurosci Lett 262: 159–162. 10218880 10.1016/s0304-3940(99)00064-6

[36] Aiello LP, Avery RL, Arrigg PG, Keyt BA, Jampel HD, et al (1994) Vascular endothelial growth factor in ocular fluid of patients with diabetic retinopathy and other retinal disorders. N Engl J Med 331: 1480–1487. 7526212 10.1056/NEJM199412013312203

[37] Tufro A, Veron D (2012) VEGF and podocytes in diabetic nephropathy. Semin Nephrol 32: 385–393. 22958493 10.1016/j.semnephrol.2012.06.010 PMC3438453

[38] Holmes K, Roberts OL, Thomas AM, Cross MJ (2007) Vascular endothelial growth factor receptor-2: structure, function, intracellular signalling and therapeutic inhibition. Cell Signal 19: 2003–2012. 17658244 10.1016/j.cellsig.2007.05.013

[39] Sondell M, Sundler F, Kanje M (2000) Vascular endothelial growth factor is a neurotrophic factor which stimulates axonal outgrowth through the flk-1 receptor. Eur J Neurosci 12: 4243–4254. 11122336 10.1046/j.0953-816x.2000.01326.x

[40] Schratzberger P, Walter DH, Rittig K, Bahlmann FH, Pola R, et al (2001) Reversal of experimental diabetic neuropathy by VEGF gene transfer. J Clin Invest 107: 1083–1092. 11342572 10.1172/JCI12188 PMC209283

[41] Pawson EJ, Duran-Jimenez B, Surosky R, Brooke HE, Spratt SK, et al (2010) Engineered zinc finger protein-mediated VEGF-a activation restores deficient VEGF-a in sensory neurons in experimental diabetes. Diabetes 59: 509–518. 19934008 10.2337/db08-1526 PMC2809974

[42] Scarlato M, Previtali SC (2011) POEMS syndrome: the matter-of-fact approach. Curr Opin Neurol 24: 491–496. 21677582 10.1097/WCO.0b013e328348e107

[43] Badros A, Porter N, Zimrin A (2005) Bevacizumab therapy for POEMS syndrome. Blood 106: 1135. 16033956 10.1182/blood-2005-03-0910

[44] Deguchi T, Hashiguchi T, Horinouchi S, Uto T, Oku H, et al (2009) Serum VEGF increases in diabetic polyneuropathy, particularly in the neurologically active symptomatic stage. Diabet Med 26: 247–252. 19317819 10.1111/j.1464-5491.2009.02680.x

[45] Mironidou-Tzouveleki M, Tsartsalis S, Tomos C (2011) Vascular endothelial growth factor (VEGF) in the pathogenesis of diabetic nephropathy of type 1 diabetes mellitus. Curr Drug Targets 12: 107–114. 20735351 10.2174/138945011793591581

[46] Abu El-Asrar AM, Nawaz MI, Kangave D, Mairaj Siddiquei M, Geboes K (2013) Angiogenic and vasculogenic factors in the vitreous from patients with proliferative diabetic retinopathy. J Diabetes Res 2013: 539658. 23671874 10.1155/2013/539658 PMC3647558

[47] Abedi F, Wickremasinghe S, Richardson AJ, Islam AF, Guymer RH, et al (2013) Genetic influences on the outcome of anti-vascular endothelial growth factor treatment in neovascular age-related macular degeneration. Ophthalmology 120: 1641–1648. 23582991 10.1016/j.ophtha.2013.01.014

[48] Cha JJ, Kang YS, Hyun YY, Han SY, Jee YH, et al (2013) Sulodexide improves renal function through reduction of vascular endothelial growth factor in type 2 diabetic rats. Life Sci 92: 1118–1124. 23643633 10.1016/j.lfs.2013.04.008

[49] Mitry D, Bunce C, Charteris D (2013) Anti-vascular endothelial growth factor for macular oedema secondary to branch retinal vein occlusion. Cochrane Database Syst Rev 1: CD009510. 10.1002/14651858.CD009510.pub2 23440840

[50] Nicholson BP, Schachat AP (2010) A review of clinical trials of anti-VEGF agents for diabetic retinopathy. Graefes Arch Clin Exp Ophthalmol 248: 915–930. 20174816 10.1007/s00417-010-1315-z

